# On the Origin of Cholera in Hindostan, and Its Conveyance to Europe

**Published:** 1868-07

**Authors:** John C. Peters

**Affiliations:** New York


### CHICAGO MEDICAL EXAMINER.

N. S. DAVIS, M.D., Editor.

VOL. IX. JULY, 1868. NO. 7.

### Original Contributions.

### ARTICLE XXII.

### ON THE ORIGIN OF CHOLERA IN HINDOSTAN, AND ITS CONVEYANCE TO EUROPE.

By JOHN C. PETERS, M.D., of New York.

It is well known that the great epidemic of 1817 commenced in the Delta of the Ganges, formed by the confluence of that huge river with the equally large Burrampooter. These immense rivers divide into innumerable branches, and empty into the Bay of Bengal, by almost as many mouths.

This land, called the Sunderbunds, lies so low that for hundreds of miles the whole country is almost a continuous swamp, intersected by numerous lagoons, from which fetid exhalations, caused by the rapid decomposition of animal and vegetable matters, rise and hang over the land like a dense fog. The sun seen through this noxious vapor loses none of its power, although it looks, even at midday, as if obscured by ground glass. The hot, damp, fetid air seems to clog and impede the free action of the lungs, and one feels instinctively that its impurities are pregnant with disease. The damp atmosphere of a hothouse, which has been shut a long time, is less oppressive and far more healthy than the normal atmosphere of the Sunderbunds. In addition, the huts of the natives are built on high artificial mounds, and cattle are frequently obliged to be housed under the same roof with the family; often in the same room. It is only rarely that the mounds are large enough for the cattle to be left outside.

Still more unfortunately, throughout the whole of the year 1816, low fevers predominated throughout this entire district, because there had been no cold and dry season as usual, and naught but mists, fogs, rains, and sultry winds prevailed. The mortality in 1816 surpassed anything on record in the annals of Bengal. The English military stations wore a gloom hardly to be imagined. In many native villages the whole population was ill, and the shops were shut for want of people to attend them. The banks of the rivers were at all times covered with the dead and dying; many bodies were left unburied in the towns; and throughout Upper Bengal (near Patna?) the horned cattle were so sickly that their carcasses could be seen strewn in vast numbers in the pastures.

Common cholera-morbus, which has always prevailed more or less epidemically in the lower provinces of Bengal during the hot seasons, commenced much earlier during the first six months of 1817, and was more prevalent than in former years. In addition, one of those great religious revivals which take place among the Hindoos every twelfth year, fell upon the year 1817.

It is now well known that all junctions of streams, all places where great rivers empty into the sea, or where the waters of two seas mingle, are held in great veneration by the Hindoos, who make huge pilgrimages to them at least once a year. The principal holy places in the Delta of the Ganges are Songar Island, at the mouth of the Hooghly, just below Calcutta; the islands at the mouths of the Brahmapootra, where it divides into the so-called Megna and Hattia rivers; the junction of the Ganges and Brahmapootra rivers, at or near the great city of Dacca; Kishnagar, at the junction of the Ganges and Jellinghy rivers, and only 70 miles north of Calcutta. Further up the river the remarkable mountain Junghera rises like an island from the majestic Ganges, and was formerly considered the holiest spot along the whole course of the river, so that thousands of boats and larger vessels were constantly to be seen there, as many Hindoos thought they could not die in peace without visiting it. Still further north, at the great city of Patna, a fair is held annually, which attracts a vast concourse of people to its festivities. A short distance south of Patna is Gaya, the supposed birthplace of Buddha, the scene of Vishnu's incarnations, and visited annually by vast numbers of pilgrims.

Whenever cholera is brought to, or originates at, these places, its intensity is very much increased by the filth of the innumerable pilgrims who then congregate together. Thus the extreme point of Songar Island, at the mouth of the Hooghly, is believed by some Hindoos to mark the junction of the Ganges with the sea, and they accordingly esteem it one of the holiest spots in Bengal, and flock thither every spring in vast numbers, for the purpose of bathing and offering sacrifices. There is much reason for the belief, that this island was one of the first great centres of cholera in 1817, and that the disease was carried up the Hooghly both to Calcutta and Jeypore, although both of these cities are always filthy enough to generate cholera within their own limits. At least, it is now well known that the disease was in Calcutta in the first week in August, and did not reach Jeypore, 100 miles to the north-east, till the 28th of the same month. But it was in Kishnagur, 70 miles north of Calcutta, in May and June, and may have been carried from there down to Calcutta, and over to Jeypore, which is only 50 miles east. Again, it was in the city of Patna, 350 miles up the Ganges, as early as the 11th of July, and may have originated there, or have been brought to it from Gaya or Jenghira.

We have also to mention that large numbers of the Hindoos do not regard the Hooghly River as the true Ganges, but place the mouth of this river much farther east, towards the province of Arracan and the kingdom of Birmah, and perform their pilgrimages to the islands at the mouths of the united Ganges and Brahmapootra rivers, near the great rivers Megna and Hattia. From there cholera was carried up the river to the province and city of Dacca, early in July; first appearing at Sunergong, a town on the banks of the great river Megna; thence proceeding north, visiting the ghauts, public ferries, and grain markets in its way. From Dacca, it was carried north-east to Sylhet, by the 17th of August, and reached Rungpore, high up the Brahmapootra river, towards Thibet and the western part of China, by the 15th of October.

From the united mouths of the Ganges, Burrampooter, and Megna rivers, it was also carried far around the eastern Bay of Bengal to the city of Chitagong, by the 23d of August, and from thence down to Arracan, to Rangoon in the Burman Empire, and to Bankok in Siam.

Hence we can readily agree with James Johnson, that "there are facts more than sufficient to show the fallacy of every theory which attempts to derive the disease from any one local source, or to trace it to any one particular spot as the centre from which alone it was emitted to the surrounding country." The facts prove, without the possibility of dispute, that it broke out at very remote places at nearly one and the same time, or at great distances at such intervals of time as to establish the impossibility of the pestilential virus having originated at any one place exclusively. James Johnson says, "It is clear to demonstration, that it did not originate in Jeypore alone, on August 28th. On the contrary, there is as good, and indeed better, reason to suppose that it was carried to Jeypore, which was merely the first place that was distinctly heard from. Thus there is little doubt that it visited some spots of the town of Calcutta and suburbs as early as the beginning of August; that it daily gained ground, and before the end of the month had widely spread its ravages."

The great religious festivals in Hindostan take place, some in February and March, others in April of every year. It is enough to say that epidemic cholera evidently broke out in May or June, 1817, soon after these festivals and after seasons remarkable for irregularity, and a preceding year distinguished for epidemic sickness and mortality. Near a sacred and filthy river it was born, and at the most sacred and filthy spots on this river, in the Delta of the Ganges. For rivers and filth it has ever subsequently testified a strong regard, especially for the large and dirty towns on such rivers, and for the centres of commerce, business fairs, and religious festivals.

From Patna the disease passed up the Ganges to the holy city of Benares, and from there to Allahabad, the city of God. The latter city is almost wholly given up to idolatry, and has ever been celebrated for the pilgrimages of pious Hindoos, attracted to a spot blessed by the junction of two sacred rivers, the Ganges and Jumna. It is esteemed holy by all castes, who annually repair in crowds to bathe themselves in the united streams. In former and more barbarous times, the junction of the Ganges and Jumna was the scene of fearful human sacrifices every year.

We have seen that cholera has always followed the routes to and from sacred rivers and places; but these are so numerous in Lower Bengal, that its course seemed for a long time both confused and erratic. At Allahabad, which it either reached in March, 1817, or broke out spontaneously, just after the great festival, which always takes place in February, even that stanch anti-contagionist, Dr. James Johnson, of the *Medico-Chirurgical Review*, is obliged to admit that "the epidemic began clearly to show one of its most striking peculiarities, which subsequently characterized its march. It no longer seemed to push its influence without distinction or apparent choice in all directions, and throughout every tract coming in its way; but began to affect particular lines, and to fix itself in particular divisions of the country; wholly restricting itself for the time to the course of those lines and divisions."

From Allahabad the disease passed north, along three great lines: first, along the river Jumna, up to the great cities of Delhi and Agra; second, along the Ganges, up towards its source; third, along the great trunk road, between the two rivers, through the towns of Coel and Meerut, directly towards HURDWAR. It is very interesting to notice that another stream of cholera was in all probability coming down from the great festival at Hurdwar, as the Bengal report says: "It is very curious that Muttra, situated considerably higher up the river Jumna than Delhi and Agra, should have had the disease in the beginning of June, whilst the latter place was not visited till the 1st of July. As the Punjaub, or extreme north-western province of Hindoostan, did not fall into the possession of the British till many years later, the progress of this epidemic could not be traced farther to the north. All the subsequent pestilences have been.

There is a great nursery of cholera at Hurdwar, at the source of the Ganges, near the foot of the Himalaya Mountains. From thence the disease is often carried down that river to Allahabad and other places. But it is also always carried north, through the great town of Lahore, up to Attock, where the Cabul River joins the Indus, and from there to Peshawur, the north-western border town of Hindostan. All the trade of Russia, Persia, Independent Tartary, Cabul, and Afghanistan, with Hindostan, comes to Peshawur and Attock; for these places are situated at the only break or pass in the Himalaya Mountains, for the space of over 1000 miles. 8000 men, 10,000 oxen of transport, and 30,000 camels are employed in this traffic. From Peshawur the road leads to the city of Cabul; there it divides in two: one leading due west to Herat, from there to Meshed, and thence to Teheran, where it meets the great route which comes up the Persian Gulf to Bushire, Shiraz, and Ispahan to Teheran, and thence on to Trebisond, Odessa, and Astrakan.

The other passes north-west from Cabul to Balk, and from thence to Bokhara and Khiva. From the latter place, one stream of trade and travel crosses to the Caspian Sea to Astracan; the other passes up between the Aral and Caspian Seas to Orenburg, on the Ural River, the great border town of Russia.

To return to Allahabad. We find that the great province of the BUNDELCUNDS lies due south of this city; and the great war against the Mahrattas was being conducted by the Marquis of Hastings with 90,000 men, in the year 1817. 10,000 natives had already died in the city of Allahabad, when on the 6th, 7th, or 8th of November it made its appearance in the army. "After creeping about in its wonted insidious manner for several days, among the lower classes of camp-followers, it, as it were in an instant, gained fresh vigor, and, at once, burst forth with irresistible violence in every direction. The natives, thinking their only safety lay in flight from the army, now began to desert in great numbers; and the highways and fields, for many miles around, were strewed with the bodies of those who had left the camp with the disease upon them, and speedily sunk under its exhausting effects. The line of march of the army presented a most deplorable spectacle. Although most, even of the ammunition carts, were used as ambulances, the sick were soon too numerous to be moved, and the greater part were left behind to die unattended. Many who left the carts, pressed by the sudden calls of the disease, were unable to rise again, and were abandoned. Hundreds dropped down during every subsequent day's advance, and covered the roads with the dead and dying. The places of encampment and the lines of march presented the appearance of a field of battle and the track of an army retreating under every circumstance of defeat and discomfiture. In less than two weeks, nearly 9000 men succumbed to the pestilence."

While this dreadful havoc was going on, a subsidiary force was sent up from Bombay, through the towns of Poonah, Seroor, Jaulnah, Ahmednuggur, and Amungabad, to the great city of Nagpore, lying due south of Allahabad and the Bundelcunds. The disease reached Nagpore from the Marquis of Hastings' army, through the cities of Huttah and Jubbelpore, pursuing a due southerly course; and the subsidiary force coming north under Colonel Adams, afforded the first striking instance of a large body of men coming into the pestilential medium, and from the previous enjoyment of perfect health, falling, at once, into a wretched state of sickness. When nine miles south of Nagpore, it had hardly learnt that the epidemic was raging in the city and vicinity, when it began itself to experience its unwelcome visits. 70 cases and 20 deaths occurred on the first day. Many were attacked while loitering for water at the neighboring contaminated rivulets, and some were brought in expiring, others dead.

From NAGPORE, it passed down to Jaulnah, the next town in order towards Bombay, in the south-west. The course of the disease had long been so regular along the line of much-traveled roads and the marches of troops, that the Bombay authorities concluded, "when it showed itself at Jaulnah, it was pretty clear that it would reach Bombay," although many hundred miles off. It was well known that it had "reached Jubbelpore, north of Nagpore, by the 9th of April. It arrived in Nagpore in May; was carried down to Jaulnah in June, by a detachment of troops that was marching from Nagpore to Jaulnah and Aurungabad; then passing over a space of 220 miles, visiting Aurungabad and Ahmednuggar in its course, it reached Seroor, 150 miles farther to the south-west, on the 18th or 19th of July. It appeared at Poonah towards the latter end of the same month. On the 6th of August, it broke out with great violence at Pantrell, a considerable village on the main line of communication between Poonah and Bombay, and distant about 20 miles; and on the 9th or 10th of the same month, the first case in Bombay occurred in a man who had arrived from Pantrell the same day. The disease could be traced as if creeping along from town to town, village to village, and from place to place, by the arrival of people affected with the disease from places where the disease was known to prevail."

Cholera prevailed severely in 1818 and 1819 in Bombay, and then moderately for several years, but in 1821 it broke out again in its most mortal and malignant form. 800 British troops, under Captain Thompson, were sent to the Island of Kishme, at the entrance of the Persian Gulf, and from there sent in the rear of Muscat, to coöperate with the Iman of that place; but they were massacred by the Bedouins, so that only 150 escaped back to the city. 3000 more troops were sent from Bombay, under Sir Lionel Smith, and cholera was introduced by them to Muscat and carried up the Persian Gulf to Bushire and Bassorah, the only ports of any importance on that sheet of water.

Bushire is not only the principal, but the only port of Central Persia, and the communication with Shiraz is incessant. Many writers say that it was transported by travelers from the former to the latter place; and 6000 persons died of it in 18 days. From Shiraz it was carried to Yedz and Teheran, and from there to Resht, at the foot of the Caspian Sea, and from there to Astrachan.

Teheran has always been a great centre for the distribution of the disease. It was carried both from Teheran and Ispahan to Kasbin, in July, 1822, to Tauris in September, and extended immediately to Erzeroun, and from there to Trebisond, on the Black Sea.

Bassorah is, also, the principal and only port at the head of the Persian Gulf. Cholera appeared there with extraordinary violence in 1821, as from 15,000 to 18,000 persons died in 18 days. From this city, it was carried by boats and caravans up the river Tigris to the great city of Bagdad, which became infected, together with the surrounding country. A Persian army at this time menaced Bagdad, and defeated the Turkish force which was collected for its defence. A few days after the victory, the Prince Royal of Persia saw his army devastated by the epidemic, and recoiled before this new enemy; but carried the disease into the centre of Persia from this new direction. He lost 2000 soldiers in one march, and when the army reached Kasbin and Tauris, from 30 to 40 were dying each day.

Thus cholera was introduced into Tauris both from Teheran, Ispahan, and Bagdad, and one-half of the whole population, or 4800 persons, died in 25 days.

From Tauris, the disease was carried up between the Black and Caspian Seas to Tiflis, and again to Astrakhan, where it had already arrived from Teheran and Resht.

From Bassorah, the epidemic also extended up to Aleppo and Damascus, near the Mediterranean coast.

Every successive epidemic of cholera has always been carried from Bombay up the Persian Gulf, and thus reaches the Mediterranean, Black, and Caspian Seas.

Trebisond is the only port at the south-eastern end of the Black Sea, as the whole eastern shore is shut in by high mountains. The trade and travel of Persia to the Black Sea must go there, and cholera always follows in their train. From Trebisond the disease has frequently been carried both to Odessa and Constantinople. This was the case in 1830, and notably so in 1847, when the first case in Constantinople occurred in a passenger from a ship just arrived from Trebisond, where cholera and cholerine prevailed. In 1854 and 1865, the disease reached Constantinople first, and was carried from thence to Trebisond.

From Odessa, the disease has repeatedly been carried west to Vienna, and thus distributed to South Germany.

It is well known how often cholera has been carried up the River Volga to Moscow, and from thence distributed to Riga, the principal Russian port on the Baltic, and to St. Petersburg. It is also well known how often the little Moravian town of Sarepta has escaped the disease, in consequence of strict quarantine and great cleanliness. Five epidemics of cholera have passed by it, and none has ever entered its streets, although it is situated in the most dangerous place imaginable, *viz.*, near the great elbow which the River Volga makes towards the west and approaches very near the River Don, which makes an equally large curve towards the east, so that the two great rivers touch each other very nearly. The trade and travel between the Don and Volga are very great at this spot, and the disease is always carried over from the Volga to the Don at this place, yet Sarepta always escapes. In 1830, over 40,000 Don Cossacks died in the small corner embraced by the lower ends of these two rivers, yet Sarepta saved herself.

In 1831, a great Polish insurrection took place, and many battles were fought in the neighborhood of Warsaw. Don Cossacks carried the disease through Kier to Warsaw; another stream of cholera came up from Odessa; and a third was conveyed from Moscow, through Smolensk to the rear of Warsaw, and carried down the River Vistula to Danzig, on the Baltic. While many precautions were being taken at Danzig to prevent infection from Riga, the disease slipped in at the back door. From Warsaw, the epidemic spread west through Posen to Berlin, and from thence to Hamburg, and from there to London.

While Vienna was surrounded by a triple cordon of troops, to prevent cholera reaching it from Warsaw and Cracow in the north, it slipped in from Odessa in the east.

To return to India, we find that Bombay not only forwards cholera up the Persian Gulf, but also up the Red Sea, and notably to Jedda and Mecca. Jedda is the port of Mecca, and all the pilgrims who come by water land there; its streets are crowded with Turks, Egyptians, Arabs, Persians, Affghans, Algerines, people from Tunis and Tripoli, Hindoos, Nubians, Abyssinians, and Negroes of every shade. Crowds of poor Hindoos litter the streets like dogs; they have performed their pilgrimage to Mecca, but are destitute of means to return home. The English consul is often obliged to give 6000 free passages on merchant vessels which sail for Bombay every autumn; yet hundreds and thousands of families live in the streets until they can obtain passages; although small brigs of only 200 tons have carried as many as 270 persons. In 1831, Jedda had 10,000 tons of shipping, and as much more in large boats, engaged in this pilgrim trade, and soon collected together a more motley assemblage of human beings than is found on any other spot on the globe; 20,000 came from Egypt alone; 120 boats were employed in carrying those from Turkey and the Barbary States, through Suez to Jedda; the pilgrims from Abyssinia, Nubia, and the interior of Africa crossed the Red Sea, from the ports of Cossier, Suakin, and Massuah; 4000 came from the Persian Gulf, principally from Muscat, Bushire, and Bassorah; 2000 came from Malay; 3000 from Mocha and Southern Arabia. Besides all these, six great caravans came: one from Cairo and Suez; another from Damascus, Syria, and Asia Minor; a third from Bassorah; a fourth from Bushire, across the Persian Gulf to Bahrein, and thence through Central Arabia; a fifth from Muscat; and the sixth from Yemen. The cholera of 1829 was rightly supposed by the Arabs of Jedda to have been brought up by the Hindoos from India; but it was not until the whole multitude had assembled at Mecca that it reached its utmost violence. Over 60,000 pilgrims died, and among them the Governors of Mecca and Jedda, the Pasha of the Persian caravan, and many people of distinction. The dead were thrown by hundreds into large pits, and the road from Mecca to Jedda was strewn with the dead and dying for weeks. The disease followed the pilgrims in their return passage up and down the Red Sea, and notably so to Yembo, Suez, and Cairo, which were attacked successively, as the pilgrims arrived at them. Lieut. Wellstadt, who spent five years on the Red Sea in English government surveying ships, found the halting-places of the pilgrim boats covered with dead bodies and graves. This is the way the disease always reaches Suez and Cairo, and from thence is carried to Alexandria on the Mediterranean coast. Alexandria is now the great centre and distributing port for the conveyance of cholera to Europe. From there it is carried to Beyrout, in Syria; to Smyrna, in Asia Minor; to Constantinople, in Turkey; to Ancona, in Italy; to Marseilles, in France; to Southampton, in England; and to Tripoli, Tunis, Algiers, and Morocco on the northern coast of Africa, and the south side of the Mediterranean Sea.

We have seen how cholera is brought to Alexandria from Mecca, in the East; we will now show how it is distributed along the Mediterranean to the West. Almost all the English, French, Austrian, and Italian steamships which sail on the Mediterranean carry pilgrims to Alexandria from Morocco, Algiers, Tunis, Tripoli, Malta, Turkey, Southern Russia, Asia Minor, etc., etc., in time to partake in the festivities of the *Kurban Bairam* at Mecca. For this trade yields considerable profit at little cost. In 1863, as many as 10,000 pilgrims were conveyed in British ships alone, between Alexandria and one or the other ports of Northern and Western Africa. We will record the experience of one steamer stopping at Tangier, opposite to Gibraltar: "Crowds of the dusky tents of the Hadji, or pilgrims, were seen upon the beach. The next day, from dawn to sunset, large boat-loads were poured on board, with their bags of millet, cracked wheat, little cooking stoves, charcoal, and their water-bags, to the number of 2000 persons. The night came on boisterously; the skies poured down torrents of rain; the burdened vessel plunged among the waves, shipping many heavy seas, till all were drenched and soaked, and their provisions damaged. Above the howlings of the storm arose the piteous cries of the pilgrims, as the great seas broke over them.

"It is a point of religion for these pilgrims to carry no change of raiment with them, and added to the filth of their wet and unchanged garments, there was soon added the ordure of 2000 men, women, and children kept closely packed together on deck for a fortnight, with nothing provided for their relief, but an extemporized stage of planks projecting from the vessel's side, upon which few landsmen could venture, even in moderately calm weather. The constant wash of the rain and sea alone carried away much of the filth, which, otherwise, would have been unbearable. Small-pox broke out, and several deaths occurred before arrival at Malta, but passengers and sailors combined to deceive the captain, from fear that he would report it and have his ship quarantined. The deaths are comparatively few on the voyage out, but on the return passage, when all are exhausted and worn out, as many as one-third have been known to die. Then their companions push them into the sea the moment they cease to breathe, if they can do so unobserved; or else cover over their bodies and sit upon them, like bags, until a convenient opportunity occurs.

"Every evening, when the weather permitted, prayers were said, and short passages from the Koran recited. First, the pilgrims stood unshod, bowing together, then dropping upon their knees, pressed their foreheads upon the deck in such lowly attitudes that it was difficult to resist the impression that every creature of them was not reverently humbling himself in the dust before his Creator. Finally, one evening, when the sun was was set, every face was found intently turned in one direction, and the heavens were searched by keenly peering eyes; soon, a slight thread-like arc of faintest silvery light marked the appearance of the new moon, with the arrival of which the "*Kurban Bairam,*" or *Feast of Sacrifices,* begins. Shouts, clapping of hands, and the gleaming brightness of every eye announced that all had seen the sight, which marks the anniversary of the time when Abraham attempted to offer up Isaac, or, as the Mohammedans believe, his first-born, Ishmael, their great progenitor; and when a ram was miraculously supplied and sacrificed in his stead. It is the commemoration of this event which draws such crowds to Mecca every year; and notably, every twelfth year.

For at Mecca is situated the well Zem Zem, which Hagar and Ishmael found when fainting with thirst in the wilderness, and thus saved the lives of the founders of the Arab and Mohammedan races. Abraham, it is also claimed, paid annual visits to Hagar at Mecca, on this anniversary, up to the time of her death, notwithstanding the reluctance and jealousy of Sarah.

